# Iron Overload, Microbleeding and the Role of Bilirubin in Alzheimer’s Disease Brain: Revisiting the Vascular Hypothesis

**DOI:** 10.3390/ijms26073060

**Published:** 2025-03-27

**Authors:** Eleonora Ficiarà, Rosita Rabbito, Fausto Roveta, Elisa Rubino, Innocenzo Rainero, Caterina Guiot, Silvia Boschi

**Affiliations:** 1 School of Pharmacy, University of Camerino, 62032 Camerino, MC, Italy; eleonora.ficiara@unicam.it; 2 Department of Neurosciences, Università degli Studi di Torino, 10125 Torino, TO, Italy; rosita.rabbito@unito.it (R.R.); fausto.roveta@unito.it (F.R.); elisa.rubino@unito.it (E.R.); innocenzo.rainero@unito.it (I.R.); caterina.guiot@unito.it (C.G.)

**Keywords:** Alzheimer’s disease, vascular dementia, bilirubin, iron metabolism, blood–brain barrier, oxidative stress, neuroinflammation, microbleeding

## Abstract

Alzheimer’s disease (AD) and vascular dementia (VaD) are the two most prevalent forms of dementia, sharing overlapping clinical features yet distinct pathophysiological mechanisms. While AD is primarily driven by amyloid-beta (Aβ) plaques and tau neurofibrillary tangles, VaD results from cerebrovascular pathology, including ischemic lesions and chronic hypoperfusion. However, accumulating evidence suggests that vascular dysfunction is a crucial contributor to both conditions, bridging neurodegenerative and cerebrovascular pathologies. In this review, we explore the interplay between AD and VaD, focusing on shared pathways such as blood–brain barrier (BBB) breakdown, neuroinflammation, and microvascular damage. Notably, cerebral microbleeds have emerged as a common feature in both AD and VaD, further linking vascular pathology to neurodegeneration. Microbleeding contributes to BBB disruption, iron deposition, and exacerbated oxidative stress, creating a vicious cycle that accelerates cognitive decline. We highlight the role of iron dysregulation as a key driver in AD, exacerbating Aβ accumulation, tau hyperphosphorylation, and ferroptosis. Conversely, bilirubin emerges as a molecule with theranostic potential, acting as both a biomarker and a neuroprotective agent due to its antioxidant and anti-inflammatory properties. Despite its protective role, bilirubin’s dysregulation under pathological conditions may contribute to oxidative damage and neurovascular dysfunction. In this context, the accumulation of iron from recurrent microbleeds may further disrupt bilirubin homeostasis, amplifying oxidative injury and inflammation. We propose a vascular hypothesis that integrates iron metabolism and bilirubin homeostasis, suggesting that their imbalance plays a central role in AD pathogenesis and worsening. Understanding the intricate molecular interplay between neurodegeneration and vascular dysfunction could provide novel insights into targeted interventions aimed at mitigating cognitive decline. Finally, we discuss the potential of bilirubin-based therapeutic strategies, including its role in counteracting oxidative stress and modulating neuroinflammatory pathways, offering promising avenues for future research and precision medicine in dementia.

## 1. Introduction

### 1.1. Interplay Between Alzheimer’s Disease and Vascular Dementia: Shared Pathways and Distinctions

Alzheimer’s disease (AD) is the most common form of neurodegenerative dementia, accounting for approximately 60–80% of cases globally [1]. AD is characterized by a progressive decline in cognitive functions, including memory, language, and executive abilities, severely impacting patients’ daily living activities [2]. At the neuropathological level, hallmark features include amyloid plaques and neurofibrillary tangles composed of hyperphosphorylated tau protein, leading to neuroinflammation and neuronal loss [3]. Amyloid plaques arise from the aggregation of beta-amyloid (Aβ) peptides, by-products of amyloid precursor protein (APP) cleavage, which disrupt synaptic communication and induce cell death via oxidative stress and mitochondrial dysfunction [4]. Concurrently, tau pathology involves intracellular accumulation of hyperphosphorylated tau, forming tangles that impair axonal transport and exacerbate neurodegeneration [5]. Both amyloid and tau pathologies trigger inflammatory responses in glial cells, amplifying neuronal damage [6]. The pathological progression of AD typically begins in the hippocampus and subcortical nuclei, spreading to the temporoparietal and frontal regions before encompassing the entire brain.

Vascular dementia (VaD) is the second most prevalent form of dementia after AD, resulting from cerebrovascular conditions that impair cerebral blood flow, such as stroke, atherosclerosis, and small vessel disease. These pathologies lead to ischemic lesions, white matter degeneration, and subsequent cognitive decline [7]. Unlike AD, which is defined by amyloid plaques and tau tangles, VaD’s neuropathology primarily involves structural and functional vascular damage, resulting in hypoperfusion and neuronal injury [8]. Ischemic insults in VaD present with diverse patterns, ranging from extensive cortical infarction to mild subcortical hypoperfusion. The severity of tissue damage varies, with some cases exhibiting partial reversibility and others progressing to irreversible neuronal loss driven by oxidative stress. This heterogeneity highlights the complex interplay of vascular mechanisms in dementia.

The clinical progression of VaD is unpredictable, with cognitive decline influenced by stroke type and severity. In contrast, AD follows a more uniform trajectory, characterized by progressive neuronal loss and cognitive deterioration over time. VaD typically results in a shorter life expectancy than AD, with an average survival of approximately five years post-diagnosis [7]. Neuropsychological profiles of VaD and AD overlap, complicating differential diagnosis. Both disorders share deficits in memory, language, visuospatial abilities, and executive function, though VaD may exhibit greater motor impairment. Mixed dementia, involving both AD and cerebrovascular pathology, becomes increasingly common with age, but formal diagnostic criteria remain absent. Stroke significantly increases dementia risk, with approximately 30% of stroke survivors developing cognitive dysfunction within three years (post-stroke dementia) [9]. This risk is not confined to older individuals; up to 50% of younger stroke patients exhibit cognitive deficits a decade after the initial event.

The lack of definitive diagnostic standards for distinguishing AD from VaD underscores the challenges in clinical practice. Neuropsychological tests achieve only moderate accuracy (up to 77%) in differentiating the two disorders. Specific features, such as early-onset depression in VaD and wandering behavior in AD, provide limited discriminatory value. Shared risk factors, including the apolipoprotein E *ε*4 allele, further complicate differentiation. Moreover, VaD diagnostic sensitivity and specificity vary widely depending on the criteria used (sensitivity: 20–60%; specificity: 26–80%) [7]. While AD-related cognitive decline is closely tied to disease duration, VaD progression reflects the heterogeneity of cerebrovascular damage. For instance, sudden severe dementia may follow a large cortical stroke, whereas subcortical arteriosclerosis progresses insidiously, often mimicking AD. Consequently, staging VaD relies more on dementia duration than cognitive severity, though the latter remains central to evaluating AD progression. A comparison of AD and VaD highlights their key differences in etiology, neuropathology, and clinical progression and their shared characteristics. Table 1 below displays the overlapping symptoms and challenges in differential diagnosis.

### 1.2. Cerebral Microbleeds: Pathophysiological Insights into AD and VaD

Early on, in 2008, Jonathan Stones proposed the hypothesis that capillary microhemorrhages could serve as the initial trigger for senile plaque formation in AD and related dementias [10]. He suggested that hemoglobin released during microbleeds binds to Aβ, inducing its oligomerization and subsequent self-assembly into protofibrils, fibrils, and plaques. This hypothesis is supported by evidence that microbleeds are a common occurrence in the aging brain and increase in frequency with age and dementia. Plaque formation has been observed at microbleed sites, where local ischemia and neutrophil infiltration exacerbate damage. Ischemia triggers hypoxia-inducible gene expression and the accumulation of oxidative damage metabolites, while hemoglobin specifically interacts with Aβ to promote oligomer formation. These processes collectively contribute to cognitive decline, with age-related vascular fragility and oxidative stress driving the progressive formation of microbleeds and plaques.

In 2012, Orehek introduced the “Micron Stroke Hypothesis” of AD and dementia, proposing that cumulative damage from numerous micron strokes underlies AD pathogenesis [11]. His theory was based on the anatomical organization of brain capillaries, which form metabolic units rather than arborized terminal branches. These microstrokes cause parenchymal injury that is often clinically undetectable or invisible on MRI. However, when the damage accumulates into hundreds or thousands of microstrokes, it manifests as white matter changes and cortical atrophy, hallmark features of dementia.

The role of microvascular damage in AD has been further corroborated by Schrag et al., who identified cerebral amyloid angiopathy (CAA) in 95% of AD patients [12]. Aβ, known to accumulate not only in brain parenchyma but also in cerebral blood vessels, exerts vasoconstrictive effects and directly damages the cerebrovascular system. CAA is increasingly recognized as a key link between vascular risk factors, VaD, and AD. Franzbau et al. also highlighted this connection, describing vascular damage as a persistent pathology common to both AD and traumatic brain injury [13].

Cerebral microhemorrhages are increasingly recognized as critical contributors to the pathogenesis of AD and ischemia-related dementias. Post-ischemic brain damage disrupts the BBB, increasing its permeability and allowing neurotoxic substances, including Aβ, to infiltrate the brain parenchyma. This facilitates chronic neuroinflammation, mechanical tissue destruction, and the formation of Aβ plaques. Furthermore, ischemia with subsequent reperfusion exacerbates the vulnerability of neuronal, glial, and endothelial cells to Aβ toxicity.

Cerebral hypoperfusion is a preclinical condition and has been identified as one of the most reliable predictors of individuals progressing to AD [14].

The neuropathogenesis of ischemia-related dementia unfolds in two distinct stages. Initially, ischemic neurons undergo necrosis and apoptosis. Subsequently, chronic BBB dysfunction promotes Aβ leakage into the brain, driving plaque formation and additional neuronal death. Repeated sub-lethal ischemic events or silent microstrokes contribute to the gradual cognitive decline characteristic of AD. These findings underscore the multifaceted role of vascular damage and microhemorrhages in linking AD and vascular dementia.

### 1.3. Vascular Comorbidities and Their Role in Cognitive Impairment

The global increase in dementia, largely driven by an aging population, is closely associated with vascular risk factors such as hypertension, diabetes, and atrial fibrillation. These conditions not only lead to vascular dementia but also elevate the risk of AD by fostering the accumulation of neurotoxic proteins in the brain.

#### 1.3.1. Hypertension

Hypertension contributes to cognitive deficits by inducing structural and functional damage to cerebral blood vessels and by directly altering central nervous system (CNS) functions through modifications of the brain’s renin–angiotensin system (RAAS). Notably, the CNS possesses an intrinsic RAAS that regulates key physiological processes essential for brain function. Chronic hypertension during midlife—specifically in the 40s and 50s—has been strongly correlated with a significant increase in the risk of cognitive impairment and dementia two decades later [15].

Hypertension promotes AD progression through multiple mechanisms, including Aβ deposition, microhemorrhages, and cognitive decline. Angiotensin II (AngII) activation is implicated in the upregulation of beta and gamma secretases, enzymes responsible for Aβ production, and in the disruption of Aβ clearance across the (BBB). Furthermore, hypertension activates the receptor for advanced glycation end products (RAGEs), which facilitates Aβ accumulation and exacerbates cognitive deficits.

In addition to these molecular pathways, hypertension induces cerebrovascular changes that include structural remodeling, reduced vascular compliance, and impaired cerebral autoregulation. These changes disrupt the perivascular spaces essential for clearing neurotoxic waste products from the brain. Such dysfunction can hinder the removal of Aβ and tau proteins, two hallmarks of AD pathology. Moreover, atherosclerosis, common in hypertension, increases vascular pulsatility, impairing interstitial fluid (ISF) and cerebrospinal fluid (CSF) clearance via the glymphatic system. The resultant accumulation of neurotoxic metabolites further exacerbates neuronal damage and cognitive decline [16].

The impact of hypertension extends beyond structural damage, influencing overall cognitive performance. Individuals with vascular risk factors, including hypertension, consistently score lower on cognitive assessments such as the Mini-Mental State Examination (MMSE) and the Montreal Cognitive Assessment (MoCA). Hypertension particularly affects executive functions, motor speed, and attention, often correlating with the subcortical damage characteristic of VaD. Memory impairment, another significant feature in hypertensive individuals, may reflect underlying AD pathology. Mixed dementia, encompassing both AD and VaD features, presents even greater cognitive decline due to the synergistic effects of cerebrovascular damage and AD-related changes. However, human studies exploring this interplay remain inconclusive.

Biomarkers, such as Aβ and tau in blood or CSF, have yet to demonstrate reliable predictive value for identifying hypertensive individuals at risk of dementia. This gap underscores the urgent need for further research to refine diagnostic tools and elucidate underlying mechanisms [17].

#### 1.3.2. Diabetes Mellitus

Diabetes mellitus (DM), particularly type 2 diabetes mellitus (T2DM), is characterized by chronic hyperglycemia due to pancreatic β-cell dysfunction, which contributes to metabolic disturbances and systemic complications. Emerging evidence highlights molecular parallels between T2DM and AD, with insulin resistance serving as a common denominator. Impaired insulin signaling in the brain, reduced GLUT4 expression, and deficits in insulin-like growth factor (IGF) pathways are central to the shared pathophysiology. These disruptions exacerbate Aβ aggregation, tau hyperphosphorylation, and neuroinflammation, thus fostering AD progression [18,19].

Hyperglycemia, advanced glycation end products (AGEs), and their interaction with receptors for AGEs (RAGEs) amplify oxidative stress, inflammation, and neuronal damage, further linking T2DM to AD. Additionally, amylin aggregates from β-cell stress may propagate proteostasis imbalance via exosome-mediated transfer, implicating peripheral metabolic dysregulation in neurodegeneration. Most brain insulin originates from circulating pancreatic insulin, crossing the BBB via specific transporters [20]. Insulin levels in CSF are approximately 25% of the plasma levels, correlating with peripheral insulin. Aging, insulin resistance, and AD reduce insulin transport to the brain due to decreased BBB transporter expression. Insulin receptors are distributed in neurons and glial cells in brain regions critical for metabolism and cognition, such as the hypothalamus, hippocampus, cerebral cortex, cerebellum, and olfactory bulb. Similarly, IGF-1 and IGF-2 receptors are highly expressed in areas like the hippocampus and thalamus, where insulin and IGF signaling pathways overlap.

Clinical findings underscore the impact of altered brain insulin signaling on Aβ and tau dynamics. Insulin resistance impairs Aβ clearance and promotes tau aggregation via kinases such as GSK3β, perpetuating neurofibrillary tangle formation. The interplay of chronic inflammation, mitochondrial dysfunction, and impaired autophagy aggravates these processes, creating a vicious cycle of neurodegeneration [18,19].

Recent studies have also highlighted the therapeutic potential of antidiabetic agents, including metformin and GLP-1 receptor agonists, in reducing dementia risk and improving cerebral glucose metabolism in AD. These treatments not only enhance systemic insulin sensitivity but may also help modify the course of AD by targeting shared molecular mechanisms [21]. Ongoing research aims to elucidate the mechanisms underlying these effects, as well as the roles of brain insulin resistance and BBB dysfunction in AD progression.

#### 1.3.3. Atrial Fibrillation

Atrial fibrillation (AF) is the most common cardiac arrhythmia globally and has been increasingly recognized as a significant risk factor for cognitive impairment, AD and VaD. The connection between AF and dementia is multifaceted and involves hemodynamic changes, cerebral hypoperfusion, and an increased risk of microembolization, all of which can contribute to neurodegeneration and vascular damage [22,23]. Studies have shown that AF-related strokes significantly elevate the risk of subsequent cognitive decline, with patients often exhibiting accelerated progression toward dementia compared to those with non-AF strokes [24]. Additionally, the chronic inflammatory state and oxidative stress associated with AF may exacerbate Aβ deposition and tau protein pathology, hallmarks of AD [25]. In this context, elevated levels of homocysteine (Hcy) have been identified as an independent risk factor for both AD and VaD, contributing to neurotoxicity through multiple mechanisms, including endothelial dysfunction, oxidative stress, and excitotoxicity [26,27]. Increased Hcy levels are associated with BBB disruption, impaired cerebral perfusion, and heightened vulnerability to ischemic and hemorrhagic damage, further bridging AF-related vascular pathology with cognitive decline [28,29]. Moreover, recent findings suggest that hyperhomocysteinemia may interact with metal toxicity, exacerbating oxidative stress and neurodegenerative processes in aging-related diseases, including AD and VaD [30].

Conversely, the use of anticoagulation therapy in AF patients has been shown to reduce the risk of dementia, particularly in those treated with non-vitamin K oral anticoagulants (NOACs) due to their favorable safety and efficacy profiles [31]. Furthermore, strategies aimed at lowering Hcy levels, such as B vitamin supplementation (folate, B6, and B12), have been proposed as potential interventions to mitigate cognitive decline, though their efficacy remains a topic of ongoing debate [32,33].

Understanding the interplay between AF and cognitive disorders is critical for early intervention strategies aimed at mitigating the dual burden of cardiovascular and neurodegenerative diseases.

Over the years, multiple hypotheses have been proposed to understand the complex mechanisms underlying AD. Hypotheses about AD such as amyloid cascade, tau hyperphosphorylation, neuroinflammation, oxidative stress, mitochondrial dysfunction, cholinergic, and vascular hypotheses are not mutually exclusive, and all of them play a certain role in the development of AD [14]. Instead, they likely interact and influence one another in a complex network of events that culminate in AD [14].

In particular, the vascular hypothesis highlights the impact of cerebrovascular factors, including AF and hyperhomocysteinemia, on AD pathogenesis, reinforcing the role of systemic and metabolic contributors in neurodegeneration [30,34].

## 2. Microvascular Alterations in the Aging Brain

### 2.1. Neurovascular Unit and Microbleeding

Vascular risk factors such as hyperglycemia and hypertension are known to exacerbate BBB dysfunction and damage the neurovascular unit (NVU) during aging, further contributing to Aβ accumulation and chronic neuroinflammation. The concept of NVU (and of neurovascular coupling) emerged in 2001 (formalized at the 2001 Stroke Progress Review Group meeting of the National Institute of Neurological Disorders and Stroke), and attempted to provide a model connecting the activation of neurons, the related abrupt energy consumption, and the hemodynamic response enhancing the cerebral blood flow (CBF) to enable the required blood supply [35].

While neurons are mainly responsible for the activation of the machinery, the main ‘effectors’ are the glia cells and the pericytes.

*(i)*  *Microglia and astrocytes*

The deep white matter is vulnerable to decreases in both CBF and oxygen, since the vasculature that supplies the subcortical white matter descends from the surface of the cortex, making perfusion at greater depths more vulnerable.

One of its components, the microglia, is directly involved in BBB breakdown by neuroinflammation, due to the infiltration of peripheral white blood cells and by oxidative stress. It is also responsible for neurovascular uncoupling by inducing mitochondrial dysfunction in neurons, the abnormal contraction of cerebral vessels, and pericyte loss in AD [36].

In addition, microglia-mediated dysfunction of cellular components in the NVU, such as astrocytes and pericytes, can destroy the integrity of the NVU and lead to NVU impairment.

The alteration of NVU is involved in the pathological changes in AD.

Moreover, the effect of AD on glial cells (astrocytes and microglia) and neurons and their effects on the established AD biomarkers Aβ and tau were investigated [35].

In this context, astrocytes are shown to play an important part in the formation of Aβ plaques by producing the protein APOE, which has three major alleles, known as *ε*2, *ε*3, and *ε*4, with different effects on cognitive function, with *ε*4 being the most well-established genetic risk factor for late-onset AD [36], whereas *ε*2 exhibits the greatest protection and a lower risk of AD [37]. APOE *ε*2 and *ε*4 allele carriers have recently been found to show differences in cerebrovascular reactivity, with young adults carrying the APOE *ε*4 allele being found to have reduced cerebrovascular reactivity, indicating that this subgroup has impaired vascular health [38].

*(ii)*  *Pericytes*

Recent studies have shed light on the pivotal role of pericytes in maintaining NVU integrity. As a matter of fact, hypertensive episodes, microtraumatic events, etc., can mechanically affect the pericytes structure, causing BBB microdisruptures.

Capillaries in AD brains are constricted by pericytes, which causes a decrease in the CBF [39]. Actually, a reduction in CBF is the first detectable clinical change in patients with mild cognitive impairment and AD and capillaries exhibit focal constriction, explaining why the greatest vascular resistance occurs in the capillary bed rather than in the penetrating arterioles [40].

In AD patient brains, microvessels frequently show narrowed and irregular diameters and this abnormality is accompanied by a reduced capillary bed density. Additionally, some vessels in these regions collapse, lose their endothelial cells, and cease to carry blood flow; these structures are known as string vessels [41]. These pathological features highlight the significance of pericytes in maintaining capillary integrity and regulating cerebral microcirculation.

Furthermore, pericytes are dynamic regulators of the capillary diameter and CBF, capable of constricting or dilating in response to neurotransmitters [42]. For instance, glutamate induces pericyte-mediated dilation, while blocking gamma-aminobutyric acid (GABA) receptors triggers pericyte constriction, suggesting a role for pericytes in neurovascular coupling and active CBF regulation. In vivo studies using DsRed-expressing pericytes in mice have demonstrated that capillary dilation precedes arteriole dilation, supporting the notion that capillary dilation is driven by active pericyte relaxation rather than passive responses to increased arteriole pressure [43]. Further investigations into pericyte-mediated vascular control reveal that various neurotransmitters and neuromodulators affect capillary tone [44].

Modeling papers simulating cerebrovascular reactivity [45] and further developments [46] show that functional hyperemia following neuronal activation exerts an important mechanical impact on the cerebral capillaries. In particular, Anselmino et al. [47] showed that transient cerebral hypoperfusion and hypertensive events during atrial fibrillation triggered a higher variability of the cerebral hemodynamic parameters, reaching their maximum extent at the arteriolar and capillary level.

The breakdown of the BBB is a hallmark of AD, primarily driven by the loss and detachment of pericytes, which compromises the structural integrity of the BBB and increases its permeability [48]. Pericyte degeneration disrupts the NVU, exacerbating neurovascular dysfunction in AD [35].

According to Nelson et al., pericytes also influence the BBB endothelial gene expression, conferring protection from insulin resistance, iron accumulation, oxidative stress, and amyloid deposition [49]. Pericyte degeneration can therefore explain some of the characteristics of AD progression, underlying multiple pathological features of AD progression.

Further studies highlight the vicious cycle of Aβ–pericyte interactions. Aβ impairs pericyte function, increasing BBB permeability and reducing capillary blood flow, which, in turn, exacerbates Aβ accumulation [40]. This cascade perpetuates the progression of AD, as impaired BBB integrity promotes immune cell infiltration, neuroinflammation, and reduced Aβ clearance [50]. Paradoxically, while Aβ is toxic to pericytes, these cells play a pivotal role in Aβ clearance via phagocytosis and translocation across the BBB [51].

The critical role of pericytes in maintaining NVU integrity and cerebral perfusion underscores their potential. In fact, ElAli et al. suggest that pericytes represent potential targets for NVU repair strategies, offering opportunities for therapeutic interventions aimed at restoring neurovascular function and protecting neurons in different brain pathologies [52].

### 2.2. The Fingerprints of Heme in the Brain

Heme fingerprints in the brain are complex and multifaceted, influencing a wide range of physiological processes from neuroprotection and energy metabolism to cellular signaling. While heme’s roles in the brain are crucial for maintaining homeostasis, its dysregulation can lead to neuronal damage and contribute to the pathogenesis of neurological diseases. Understanding the delicate balance of heme metabolism and its effects on brain health could provide new therapeutic targets for treating various neurodegenerative and cerebrovascular disorders.

**Figure 1:**
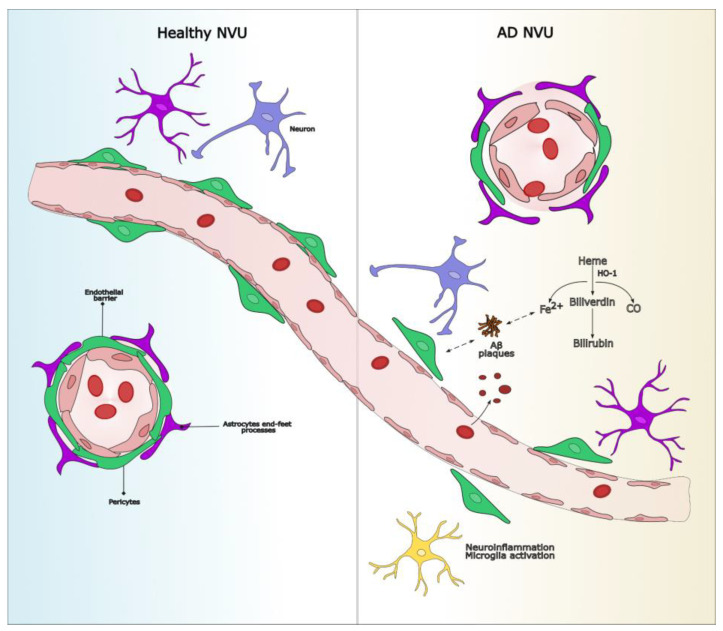
Dysfunction of the NVU in AD involves microvascular damage, BBB disruption, and neuroinflammation. In the healthy condition, the NVU regulates cerebral homeostasis through intact endothelial junctions and metabolic support from glial cells. In AD, capillary microhemorrhages release hemoglobin, which is degraded by HO-1 into iron, bilirubin, and carbon monoxide. Excess iron interacts with Aβ, promoting its aggregation into toxic plaques, leading to chronic neuroinflammation, neuronal damage, and cognitive decline. Aβ accumulation also impairs pericyte function. The dual role of bilirubin (protective versus neurotoxic) may be investigated to determine its viability as a theranostic agent. AD: Alzheimer’s disease; Aβ: amyloid-beta; BBB: blood–brain barrier; HO-1:heme oxygenase-1; NVU: neurovascular unit.

Following microbleeding events, small quantities of hemoglobin reach the brain extracellular fluid, where, in the presence of the heme oxygenase/biliverdin reductase system (HO/BVR), heme produces equimolar amounts of carbon monoxide (CO), iron(II), and biliverdin, which is immediately reduced to bilirubin (see Figure 1). Heme can therefore be indirectly detected by traces of HO, iron, and bilirubin, due to the elusive nature of CO.

### 2.3. Heme Oxygenase 1 (HO-1) in AD and in VaD

Most research has focused on the catalytic HO-1, which catalyzes the degradation of cellular heme to free ferrous iron, biliverdin, and carbon monoxide under stressful conditions. HO-1 plays a complex role in neurodegeneration, exerting both protective and detrimental effects depending on the context. HO-1 can mitigate oxidative stress by degrading heme into biliverdin (which is subsequently converted into bilirubin), CO, and iron, which, under controlled conditions, have cytoprotective properties. Astroglial induction of the Hmox1 gene by β-amyloid, pro-inflammatory cytokines, and hydrogen peroxide promotes the mitochondrial sequestration of non-transferrin iron and macroautophagy and may thereby contribute to pathological iron deposition and bioenergy failure, amply documented in AD-affected neural tissues [53].

By converting pro-oxidant heme into antioxidants, biliverdin, and bilirubin, HO-1/biliverdin reductase may help restore a more favorable tissue redox microenvironment. Contrariwise, in some cell types and under certain circumstances, heme-derived carbon monoxide and iron may amplify intracellular oxidative stress and exacerbate the disease process [54].

Experimental observations indicate that the extent of HO-1 induction may be critical because excessive heme degradation may result in toxic levels of CO, bilirubin and, more importantly, iron [55].

A recent review investigated the dual roles of HO-1 in cellular processes, highlighting both its protective effects and its toxic effects, including oxidative stress, mitochondrial damage, ferroptosis, and autophagy [56].

HO-1 and bilirubin, products of heme degradation, exert protective effects in certain brain regions by acting as antioxidants and mitigating oxidative stress [57]. In AD and mild cognitive impairment (MCI), immunoreactive HO-1 protein is over-expressed in astrocytes and neurons of the hippocampus and cerebral cortex and co-localizes to neurofibrillary tangles, senile plaques, and corpora amylacea [58].

HO-1 dysregulation is associated with brain inflammation and neurodegeneration, including Parkinson’s diseases and AD [59].

Several lines of evidence have demonstrated that upregulated HO-1 is linked to tauopathies, neuronal damage, and synapse aberrations in AD [60]. HO-1 induction in primary astroglial cultures promotes the deposition of non-transferrin iron, mitochondrial damage, and macroautophagy, and predisposes co-cultured neuronal elements to oxidative injury [61]. HMOX1 transgenic mice selectively over-express human HO-1 in the astrocytic compartment and, at 48 weeks, exhibit increased deposits of glial iron in the hippocampus and other subcortical regions without overt changes in iron-regulatory and iron-binding proteins relative to age-matched wild-types [62].

HO-1 protects against AD amyloid-β1-42-induced toxicity via carbon monoxide production [63]. HO-1 also plays a crucial role in protecting against oxidative stress in the pathogenesis of VaD. Recent studies have shown that HO-1 induction can improve cognitive dysfunction in animal models of chronic cerebral hypoperfusion, suggesting a potential neuroprotective effect [64].

In particular, the activation of the ERK/Nrf2/HO-1 signaling pathway has been associated with reduced oxidative stress and improved cognitive function in VaD models. For instance, edaravone, a well-known free radical scavenger, has been shown to attenuate oxidative damage and mitigate cognitive deficits through the activation of this pathway [65].

Additionally, compounds such as sulforaphane have demonstrated neuroprotective effects in experimental models of vascular cognitive impairment, mediated by Nrf2 activation and the subsequent upregulation of HO-1 [66].

The suppression of glial HO-1 activity by pharmacological or other means may confer neuroprotection in AD by curtailing iron-mediated neurotoxicity [53].

### 2.4. Iron’s Role in AD Pathology vs. VaD Pathology

The link between metals and AD has been studied in the last two decades, focusing on their local accumulation in brain areas critical for AD and revealing a relation between iron and AD, particularly in relation to its capacity to increase the risk of the disease through ferroptosis.

A variety of iron metabolism-related proteins have been found to be abnormally expressed in the brains of AD patients and mouse models, resulting in iron deposition and promoting AD progression, and several recent literature reviews have explored the connection between iron dysregulation and AD pathogenesis.

Early studies found that iron overload is directly proportional to cognitive decline in AD. APP and tau protein, both of which are related to AD pathogenesis, are associated with brain iron metabolism [67].

Beyond localized iron accumulation in the brain, systemic iron overload has been proposed as a potential contributor to neurodegenerative processes, including ferroptosis. The Framingham study reported that approximately 11% of elderly individuals exhibit elevated total body iron levels, raising concerns about its potential role in neurodegeneration [68]. Chronic systemic iron overload can lead to increased iron availability in the brain due to dysregulated transport across the BBB, exacerbating oxidative stress and promoting ferroptosis, a form of programmed cell death characterized by lipid peroxidation [69].

Emerging evidence highlights the role of iron dysregulation in the pathogenesis of VaD. Iron is a vital element involved in oxygen transport, mitochondrial function, and myelin formation. However, its excess accumulation in the brain, particularly in regions susceptible to vascular injury, has been associated with detrimental effects. In VaD, iron accumulation often occurs in brain regions with prior ischemic or hypoxic events. This iron accumulation exacerbates oxidative stress through the iron-catalyzed production of reactive oxygen species (ROS) [70].

Excess iron in brain tissues can amplify neuroinflammatory responses, which are central to VaD pathology, leading to further vascular and neuronal damage. Iron also plays a role in BBB breakdown, which is commonly observed in VaD. Damage to the BBB allows iron and other inflammatory mediators to accumulate within brain tissue, aggravating oxidative damage and microvascular dysfunction [71]. Furthermore, systemic iron overload may exacerbate these processes by increasing circulating free iron, which can contribute to endothelial dysfunction, systemic inflammation, and heightened vulnerability to cerebrovascular insults [72]. This progressive damage is believed to contribute to cognitive decline by further compromising the vascular system and exacerbating neuronal injury.

While iron accumulation is implicated in both AD and VaD, its role in each condition is linked to different pathological processes. In AD, iron primarily interacts with Aβ plaques, promoting oxidative stress that exacerbates neurodegeneration [73]. In contrast, in VaD, iron-related oxidative damage occurs predominantly in the context of vascular insufficiency, inflammatory responses, and BBB dysfunction, which collectively contribute to neuronal loss through ischemic pathways.

Iron accumulation, being a critical factor in neurodegeneration, prompted the development of iron nanochelating agents aimed at restoring iron homeostasis and reducing oxidative stress [74]. Preliminary studies indicate that these nanochelators could offer neuroprotective benefits by limiting iron-driven oxidative damage in the CNS [74]. Additionally, systemic iron reduction strategies, including dietary modifications and iron chelation therapies, have been explored as potential interventions to mitigate iron-induced neurotoxicity and reduce the risk of ferroptosis-associated neuronal loss [75].

Finally, it is important to mention that AD is influenced by the toxicity of various metals, such as copper toxicity, also involving a combination of environmental toxic metals that may play a part in several common neurological disorders [76,77].

## 3. Bilirubin as a Theranostic Molecule in AD

Bilirubin, a potent endogenous antioxidant, is the final product of the heme degradation pathway, catalyzed by HO and biliverdin reductase (BVR) (see Figure 1). While an inducible form of HO is upregulated in the brains of individuals with AD, it remains unclear whether bilirubin metabolism is directly activated in this context.

Bilirubin exists in two primary isoforms: unconjugated and conjugated; the main function of bilirubin in brain physiology is to exert an antioxidant function, protecting neuronal cells from ROS and free radicals [78].

Recent work has explored the dual role of bilirubin as neuroprotective and neurotoxic, essential for establishing effective therapeutic outcomes for neurodegenerative diseases by looking at its cellular mechanisms and discussing how bilirubin’s antioxidant properties can shield neurons and, in some situations, may induce oxidative stress and apoptosis [79].

Unconjugated bilirubin has lipophilic properties, meaning that it can cross the BBB, and acts as an antioxidant at low to moderate levels, but becomes neurotoxic at elevated levels, potentially damaging brain regions such as the basal ganglia and brainstem [79].

In the context of AD, increasing the level of bilirubin in the brain seems to be protective in three ways: (1) reducing redox stress; (2) reducing Aβ deposits; and (3) reverting insulin resistance [80].

In addition to its antioxidant properties, bilirubin may reduce inflammation, playing a role in controlling the production of inflammatory cytokines [81].

However, preclinical studies in rodent models have suggested that exposure to bilirubin in newborn animals is significantly involved in the pathophysiology of AD, by increasing the accumulation of Aβ and tau hyperphosphorylation [82].

Quantifying heme and its degradation products, such as biliverdin and bilirubin, in CSF can aid in the diagnosis and monitoring of neurodegenerative diseases. Altered levels of these metabolites reflect the underlying dysregulation of heme metabolism and oxidative stress [83].

Kimpara et al. measured CSF bilirubin and its derivatives, demonstrating that in AD patients, CSF bilirubin derivative levels were significantly increased compared to those in controls [84]. This increase was not attributed to enhanced BBB permeability, as the unconjugated bilirubin levels did not differ between the AD patients and controls. These findings suggest that the accumulation of bilirubin metabolites in AD brain may result from a scavenging response to chronic oxidative stress [84].

Elevated bilirubin levels have been implicated in neurotoxicity, primarily through oxidative stress, mitochondrial dysfunction, and neuroinflammation [85]. However, bilirubin’s ability to neutralize ROS suggests it may also function as a protective agent in neurodegenerative disorders.

The entry of bilirubin into the brain is influenced by various factors, including drug-mediated displacement from albumin, reduced albumin-binding capacity, increased CBF, and BBB permeability alterations [86]. In the circulation, almost all bilirubin is albumin-bound, and only its unconjugated form can cross the BBB and enter the CNS.

Bilirubin appears to distribute differentially to brain subcellular compartments and is oxidized in the brain by an enzyme localized on the inner mitochondrial membrane and found both in neurons and in glia, but appears to be more active in the latter [86]. Furthermore, bilirubin modulates astrocytic function by reducing oxidative stress and maintaining extracellular glutamate and potassium concentrations, ensuring neuronal stability [87].

A reduction in plasma antioxidants, including bilirubin, has been identified as a hallmark of AD [88]. Additionally, indirect bilirubin (IBIL) and the IBIL/albumin ratio are significantly elevated in dementia patients with Aβ deposition, suggesting a potential link between bilirubin homeostasis and AD pathology. Notably, intravenous albumin administration has shown therapeutic benefits in AD patients, further supporting the involvement of bilirubin in disease modulation [89].

Despite its physiological relevance, the impact of bilirubin and its oxidation products on the adult brain remains underexplored. Lakovic et al. examined the effects of bilirubin on white matter integrity using ex vivo brain slices, revealing detrimental structural and functional changes [90]. Beyond its role in oxidative stress, bilirubin is part of a broader redox-regulatory system involving biliverdin reductase-A (BVRA), a multifunctional enzyme that converts biliverdin to bilirubin and modulates neuroprotective signaling pathways critical for cognitive function. Dysregulation of the BVRA/bilirubin axis has been implicated in AD pathophysiology [91].

Although AD is primarily characterized by amyloid plaques and tau pathology, and VaD by ischemic changes, both conditions share common features, including BBB dysfunction, oxidative stress, and neuroinflammation. Cerebral microbleeds contribute to heme breakdown, leading to iron and bilirubin accumulation, which may exacerbate neuronal damage. Furthermore, natural compounds that modulate the heme catabolic pathway are gaining attention for their potential role in treating gut, liver, cardiovascular, and brain diseases [92]. These compounds may help balance bilirubin’s antioxidant and pro-oxidant effects, offering new therapeutic possibilities for neurodegenerative disorders. The interplay between bilirubin, inflammation, and dopaminergic signaling is another emerging area of interest, particularly concerning neurodegenerative diseases associated with chronic inflammation [93].

Clinically, elevated serum bilirubin levels have been associated with cardiovascular protection and a reduced risk of metabolic disorders, including diabetes and diabetic nephropathy. However, the serum bilirubin levels decline with age, which may contribute to the increased susceptibility to age-related diseases [94]. Low bilirubin levels have also been linked to a higher risk of neurodegenerative conditions such as AD and amyotrophic lateral sclerosis (ALS) [95].

While few studies have directly examined bilirubin’s neuroprotective role, evidence suggests that biliverdin, its precursor, can mitigate oxidative injury and reduce the infarct size in transient ischemia models [96]. Moreover, hyperbilirubinemic rat models have exhibited neuroprotection against stroke-induced neuronal damage [97].

Given bilirubin’s lipophilic nature and limited solubility, its clinical application is challenging. In contrast, biliverdin is water-soluble and non-toxic, and it is rapidly converted into bilirubin by BVR in tissues, making it a promising candidate for therapeutic intervention in neurodegenerative disorders.

## 4. Discussion and Conclusions

Iron dysregulation plays a critical role in the pathogenesis of both AD and VaD, influencing disease progression through oxidative stress, ferroptosis, and neuroinflammatory pathways. In AD, the aberrant expression of iron metabolism-related proteins leads to iron accumulation, which exacerbates APP and tau pathology, ultimately accelerating cognitive decline. Similarly, in VaD, iron overload in ischemic and hypoxic regions may contribute to neurovascular dysfunction, BBB breakdown, and increased oxidative stress, further promoting neuronal damage and cognitive impairment.

The vascular hypothesis suggests a unifying perspective that links iron overload and bilirubin’s theranostic role in neurodegenerative diseases. This hypothesis posits that vascular dysfunction, exacerbated by iron-induced oxidative damage and inflammatory responses, plays a central role in both AD and VaD pathogenesis. Iron accumulation can disrupt cerebrovascular integrity, leading to microvascular damage and cognitive decline. Microbleeds, characterized by focal hemosiderin deposits, are increasingly recognized as key contributors to neurodegeneration, exacerbating oxidative stress and inflammatory responses while further compromising BBB integrity.

Conversely, bilirubin’s antioxidant and anti-inflammatory properties could counteract some of these effects, highlighting its potential as both a biomarker and a therapeutic target. Understanding the balance between iron overload-induced damage and bilirubin’s protective effects could provide crucial insights into disease mechanisms and novel therapeutic strategies.

The interplay between iron and neurodegeneration highlights the importance of developing targeted therapeutic strategies. Modulating iron homeostasis through iron chelators, antioxidants, or regulatory proteins could provide potential avenues for disease intervention. However, challenges remain in precisely targeting iron dysregulation without disrupting essential physiological processes. Future studies should focus on elucidating the molecular mechanisms linking iron metabolism with neurodegeneration and exploring novel therapeutic approaches that restore iron balance without inducing systemic deficiencies. Therapies aimed at mitigating microbleeding-associated damage, such as agents that stabilize vascular integrity or reduce oxidative stress, should be considered in the broader context of iron-targeting strategies.

Additionally, bilirubin has emerged as a molecule with significant theranostic potential due to its antioxidative and neuroprotective properties. Despite its promise, challenges such as lipophilicity, toxicity at high concentrations, and limited solubility must be addressed to optimize its clinical application. Advances in drug delivery systems and the development of bilirubin analogs or derivatives may help overcome these barriers. Furthermore, the integration of bilirubin measurement into personalized medicine platforms, including machine learning-based diagnostics, could enhance its role in both disease monitoring and therapeutic interventions. Ongoing research into bilirubin’s dual role—protective versus neurotoxic—will be crucial in determining its viability as a therapeutic agent, particularly in neurodegenerative diseases.

Overall, the aim of this review was to summarize the complexity of iron and bilirubin metabolism related to neurovascular dysfunction, which represent key factors in neurodegeneration, offering promising yet complex targets for future research and therapeutic development. Understanding their intricate roles in AD, VaD, and other neurodegenerative conditions will be essential for advancing effective treatment strategies aimed at mitigating disease progression and improving patient outcomes. Furthermore, a deeper exploration of microbleeding as a mediator of iron-induced neurovascular damage may provide novel insights into cerebrovascular contributions to neurodegeneration, paving the way for targeted interventions that enhance vascular health and cognitive resilience.

## Figures and Tables

**Table 1:** Comparison of AD and VaD, highlighting key differences in etiology, neuropathology, and clinical progression and shared characteristics.

Aspect	Alzheimer’s Disease (AD)	Vascular Dementia (VaD)	Similarities
Prevalence	Most common dementia type (60–80% of cases globally).	Second most common dementia type.	Both are prevalent dementia types.
Etiology	Neurodegenerative; characterized by amyloid plaques and tau tangles.	Results from cerebrovascular conditions like stroke, atherosclerosis, and small vessel disease.	Both involve cognitive decline and neuronal damage.
Neuropathology	Amyloid plaques, tau tangles, neuroinflammation, neuronal loss.	Vascular damage, ischemic lesions, white matter degeneration, hypoperfusion.	Both cause neuronal injury through oxidative stress and other mechanisms.
Pathological Progression	Begins in the hippocampus, spreading to temporoparietal and frontal regions.	Varies depending on vascular events (e.g., cortical infarction, subcortical hypoperfusion).	Both involve progressive brain damage.
Cognitive Symptoms	Progressive decline in memory, language, and executive function; uniform trajectory.	Unpredictable decline, influenced by stroke type and severity.	Overlapping deficits in memory, language, visuospatial abilities, and executive function.
Motor Impairment	Less common in early stages.	More pronounced motor impairments.	Both can affect motor functions, but to varying extents.
Life Expectancy	Longer average survival after diagnosis compared to VaD.	Shorter average survival (~5 years post-diagnosis).	Both conditions reduce life expectancy.
Diagnosis	Neuropsychological tests moderately accurate; associated with wandering behavior.	Neuropsychological tests moderately accurate; associated with early-onset depression.	Differential diagnosis is challenging; both share overlapping features.
Risk Factors	Includes APOE *ε*4 allele, age, and family history.	Includes stroke, cardiovascular diseases, and shared genetic factors like APOE *ε*4 allele.	Both share common genetic and lifestyle risk factors.
Mixed Dementia	Often coexists with vascular pathology in older individuals.	Commonly overlaps with AD pathology in mixed dementia cases.	Mixed dementia reflects shared characteristics and overlapping pathologies.
Treatment Challenges	No definitive cure; focus on slowing progression and managing symptoms.	No definitive cure; focuses on managing vascular health and preventing further strokes.	Both lack curative treatments, with emphasis on symptom management.
Vascular comorbidities	Often coexists with hypertension, diabetes, and atherosclerosis, contributing to disease progression.	Strongly linked to cardiovascular risk factors such as hypertension, atrial fibrillation, and stroke history.	Both are influenced by vascular health, with overlapping risk factors exacerbating cognitive decline.
Homocysteine’s Role	Elevated homocysteine levels are associated with increased amyloid aggregation, oxidative stress, and neurodegeneration in AD.	High homocysteine contributes to endothelial dysfunction, cerebrovascular damage, and an increased risk of stroke, exacerbating VaD progression.	Elevated homocysteine is a shared risk factor, promoting neurotoxicity, vascular damage, and cognitive decline in both conditions.
